# Supplementary material for: The interactome of intact mitochondria by cross-linking mass spectrometry provides evidence for coexisting respiratory supercomplexes
Source: Mol Cell Proteomics. 2017 Dec 8;17(2):216–32. doi: 10.1074/mcp.RA117.000470 (PMC5795388; doi:10.1074/mcp.RA117.000470)
Supplement: Supplemental Data [file supp_RA117.000470_133922_0_supp_23978_fzffwf.zip › spectra_annotation/mito_spectra_annotation/38-1-6-1-10-1.pdf]

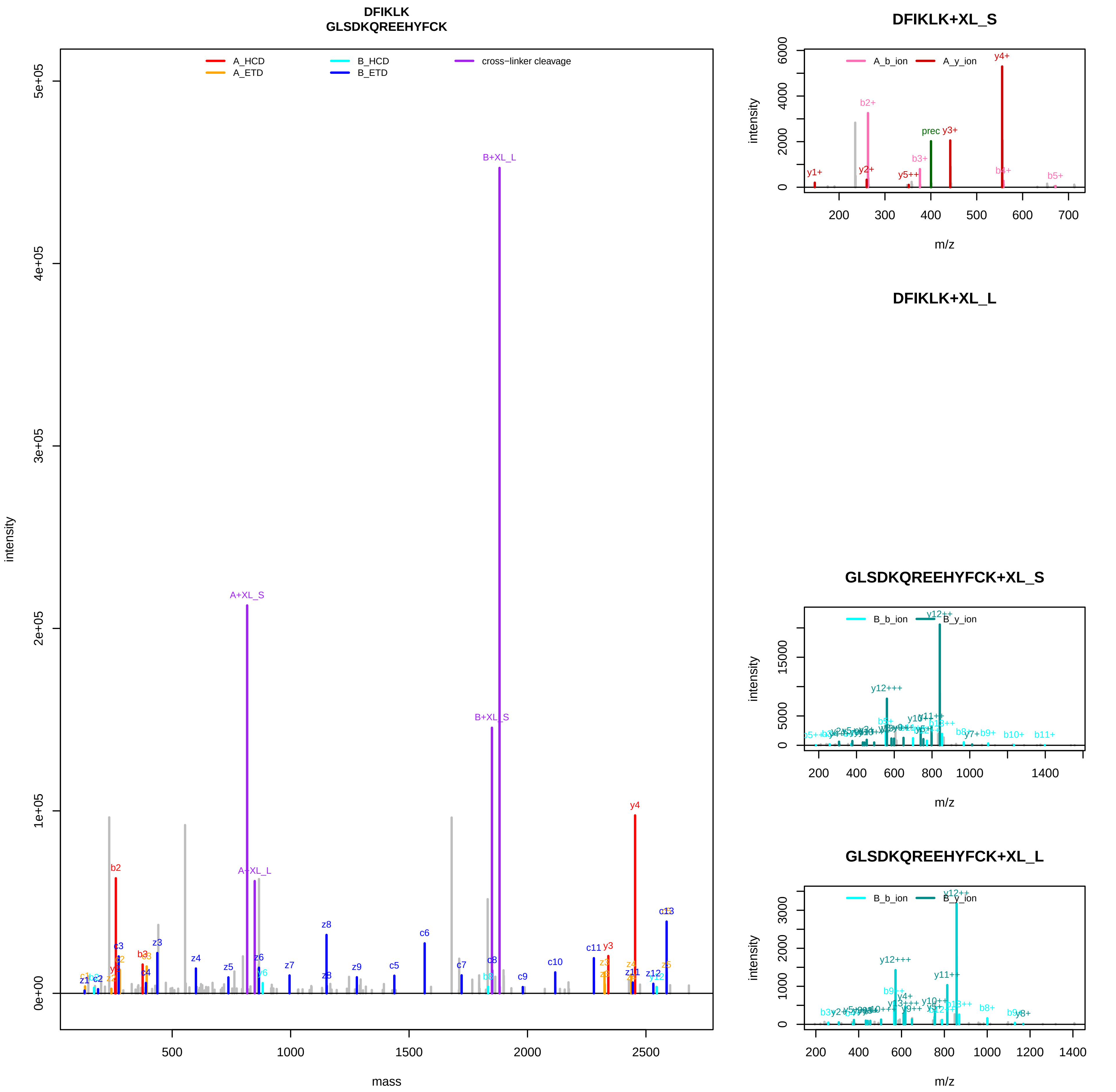
DFIKLK
GLSDKQREEHYFCK
A_HCD
A_ETD
B_HCD
B_ETD
cross−linker cleavage
B+XL_L
A+XL_S
B+XL_S
A+XL_L
y4
b2
b3
c3
z3
z4
z5
z6
z7
z8
z9
c5
c6
c7
c8
c9
c10
c11
y3
z11
z12
c13
intensity
mass
0e+00
1e+05
2e+05
3e+05
4e+05
5e+05
500
1000
1500
2000
2500
DFIKLK+XL_S
A_b_ion
A_y_ion
y4+
b2+
prec
y3+
b3+
y1+
y2+
y5++
b5+
m/z
DFIKLK+XL_L
GLSDKQREEHYFCK+XL_S
B_b_ion
B_y_ion
y12++
y12+++
y11++
b7+
y7+
b9+
b10+
b11+
GLSDKQREEHYFCK+XL_L
y12++
y12+++
y11++
b9++
y10++
b8+
y8+
y4+
